# Supplementary material for: Clinical and Biochemical Characterization of Hereditary ATTR Amyloidosis Caused by a Novel Transthyretin Variant V121A (p.V141A)
Source: Int J Mol Sci. 2025 May 13;26(10):4659. doi: 10.3390/ijms26104659 (PMC12111042; doi:10.3390/ijms26104659)
Supplement: Supplementary file 1 [file ijms-26-04659-s001.zip › supplemental_file_V121A_Yoshinaga_.pptx]

## Slide 1
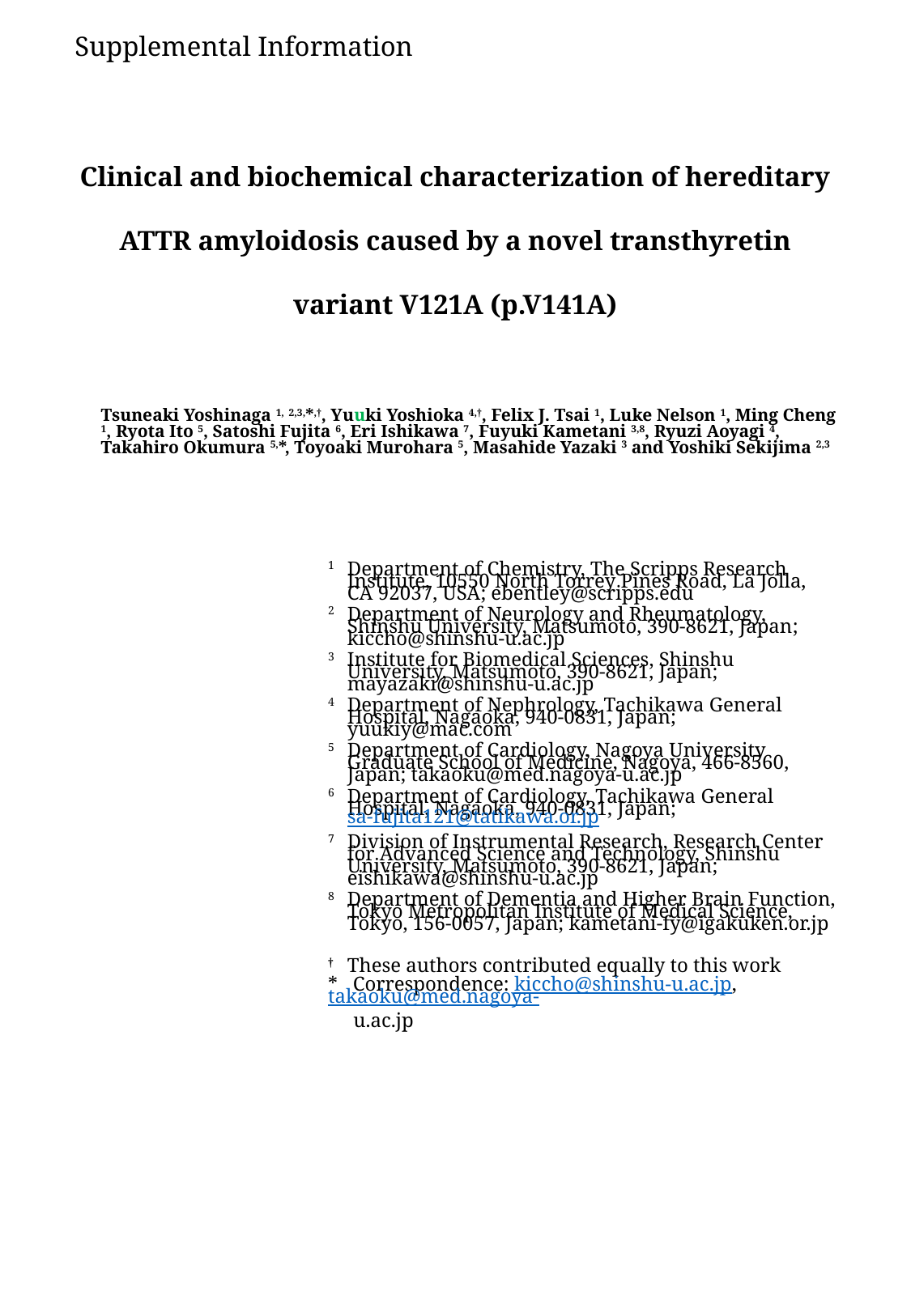

Supplemental Information
Clinical and biochemical characterization of hereditary ATTR amyloidosis caused by a novel transthyretin variant V121A (p.V141A)
 	Tsuneaki Yoshinaga 1, 2,3,*,†, Yuuki Yoshioka 4,†, Felix J. Tsai 1, Luke Nelson 1, Ming Cheng 1, Ryota Ito 5, Satoshi Fujita 6, Eri Ishikawa 7, Fuyuki Kametani 3,8, Ryuzi Aoyagi 4, Takahiro Okumura 5,*, Toyoaki Murohara 5, Masahide Yazaki 3 and Yoshiki Sekijima 2,3
1	Department of Chemistry, The Scripps Research Institute, 10550 North Torrey Pines Road, La Jolla, CA 92037, USA; ebentley@scripps.edu
2	Department of Neurology and Rheumatology, Shinshu University, Matsumoto, 390-8621, Japan; kiccho@shinshu-u.ac.jp
3	Institute for Biomedical Sciences, Shinshu University, Matsumoto, 390-8621, Japan; mayazaki@shinshu-u.ac.jp
4	Department of Nephrology, Tachikawa General Hospital, Nagaoka, 940-0831, Japan; yuukiy@mac.com
5	Department of Cardiology, Nagoya University Graduate School of Medicine, Nagoya, 466-8560, Japan; takaoku@med.nagoya-u.ac.jp
6	Department of Cardiology, Tachikawa General Hospital, Nagaoka, 940-0831, Japan; sa-fujita121@tatikawa.or.jp
7	Division of Instrumental Research, Research Center for Advanced Science and Technology, Shinshu University, Matsumoto, 390-8621, Japan; eishikawa@shinshu-u.ac.jp
8	Department of Dementia and Higher Brain Function, Tokyo Metropolitan Institute of Medical Science, Tokyo, 156-0057, Japan; kametani-fy@igakuken.or.jp
†	These authors contributed equally to this work
* Correspondence: kiccho@shinshu-u.ac.jp, takaoku@med.nagoya-
 u.ac.jp

## Slide 2
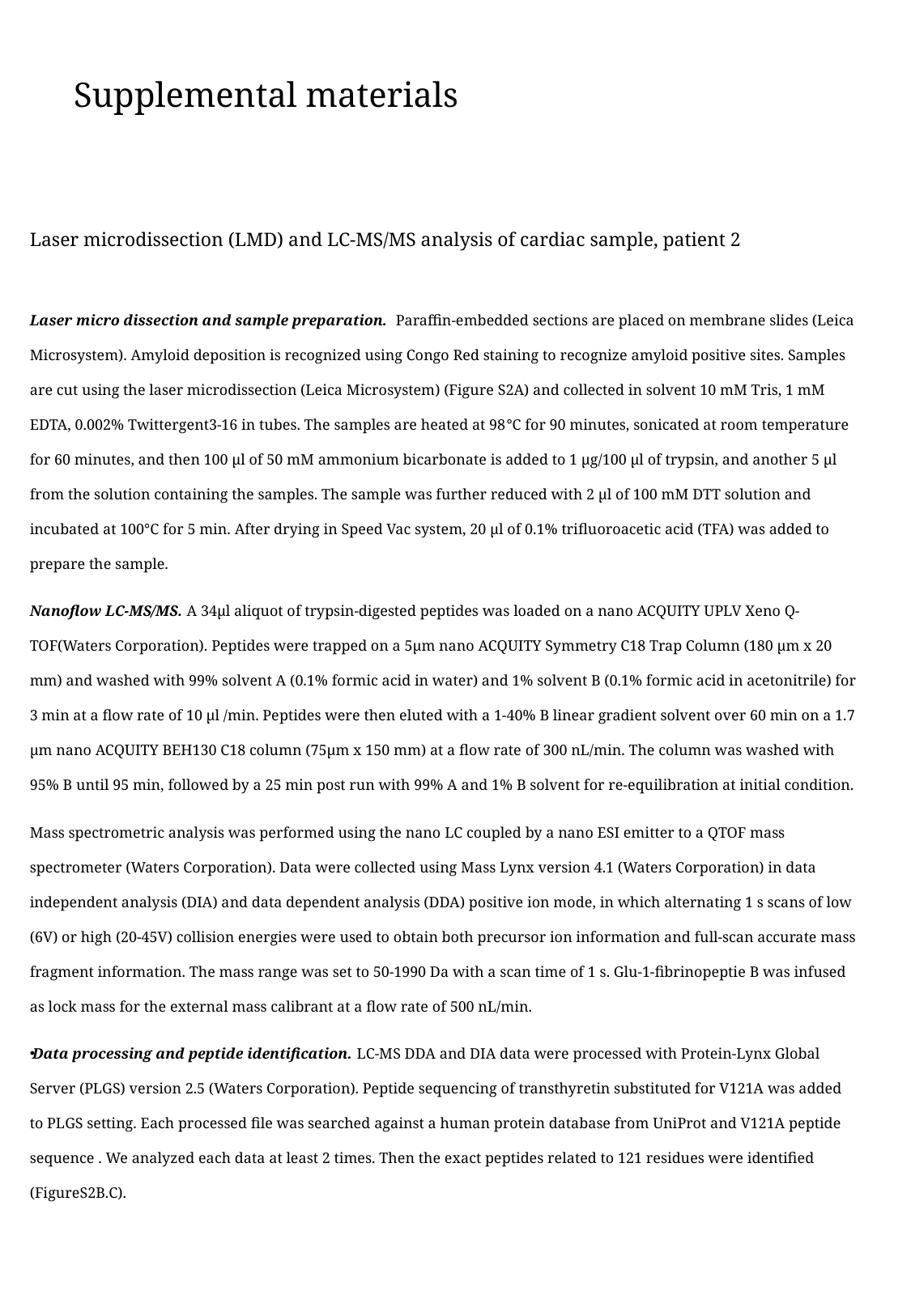

# Supplemental materials
Laser microdissection (LMD) and LC-MS/MS analysis of cardiac sample, patient 2
Laser micro dissection and sample preparation. Paraffin-embedded sections are placed on membrane slides (Leica Microsystem). Amyloid deposition is recognized using Congo Red staining to recognize amyloid positive sites. Samples are cut using the laser microdissection (Leica Microsystem) (Figure S2A) and collected in solvent 10 mM Tris, 1 mM EDTA, 0.002% Twittergent3-16 in tubes. The samples are heated at 98℃ for 90 minutes, sonicated at room temperature for 60 minutes, and then 100 μl of 50 mM ammonium bicarbonate is added to 1 μg/100 μl of trypsin, and another 5 μl from the solution containing the samples. The sample was further reduced with 2 μl of 100 mM DTT solution and incubated at 100°C for 5 min. After drying in Speed Vac system, 20 μl of 0.1% trifluoroacetic acid (TFA) was added to prepare the sample.
Nanoflow LC-MS/MS. A 34μl aliquot of trypsin-digested peptides was loaded on a nano ACQUITY UPLV Xeno Q-TOF(Waters Corporation). Peptides were trapped on a 5μm nano ACQUITY Symmetry C18 Trap Column (180 μm x 20 mm) and washed with 99% solvent A (0.1% formic acid in water) and 1% solvent B (0.1% formic acid in acetonitrile) for 3 min at a flow rate of 10 μl /min. Peptides were then eluted with a 1-40% B linear gradient solvent over 60 min on a 1.7 μm nano ACQUITY BEH130 C18 column (75μm x 150 mm) at a flow rate of 300 nL/min. The column was washed with 95% B until 95 min, followed by a 25 min post run with 99% A and 1% B solvent for re-equilibration at initial condition.
Mass spectrometric analysis was performed using the nano LC coupled by a nano ESI emitter to a QTOF mass spectrometer (Waters Corporation). Data were collected using Mass Lynx version 4.1 (Waters Corporation) in data independent analysis (DIA) and data dependent analysis (DDA) positive ion mode, in which alternating 1 s scans of low (6V) or high (20-45V) collision energies were used to obtain both precursor ion information and full-scan accurate mass fragment information. The mass range was set to 50-1990 Da with a scan time of 1 s. Glu-1-fibrinopeptie B was infused as lock mass for the external mass calibrant at a flow rate of 500 nL/min.
Data processing and peptide identification. LC-MS DDA and DIA data were processed with Protein-Lynx Global Server (PLGS) version 2.5 (Waters Corporation). Peptide sequencing of transthyretin substituted for V121A was added to PLGS setting. Each processed file was searched against a human protein database from UniProt and V121A peptide sequence . We analyzed each data at least 2 times. Then the exact peptides related to 121 residues were identified (FigureS2B.C).

## Slide 3
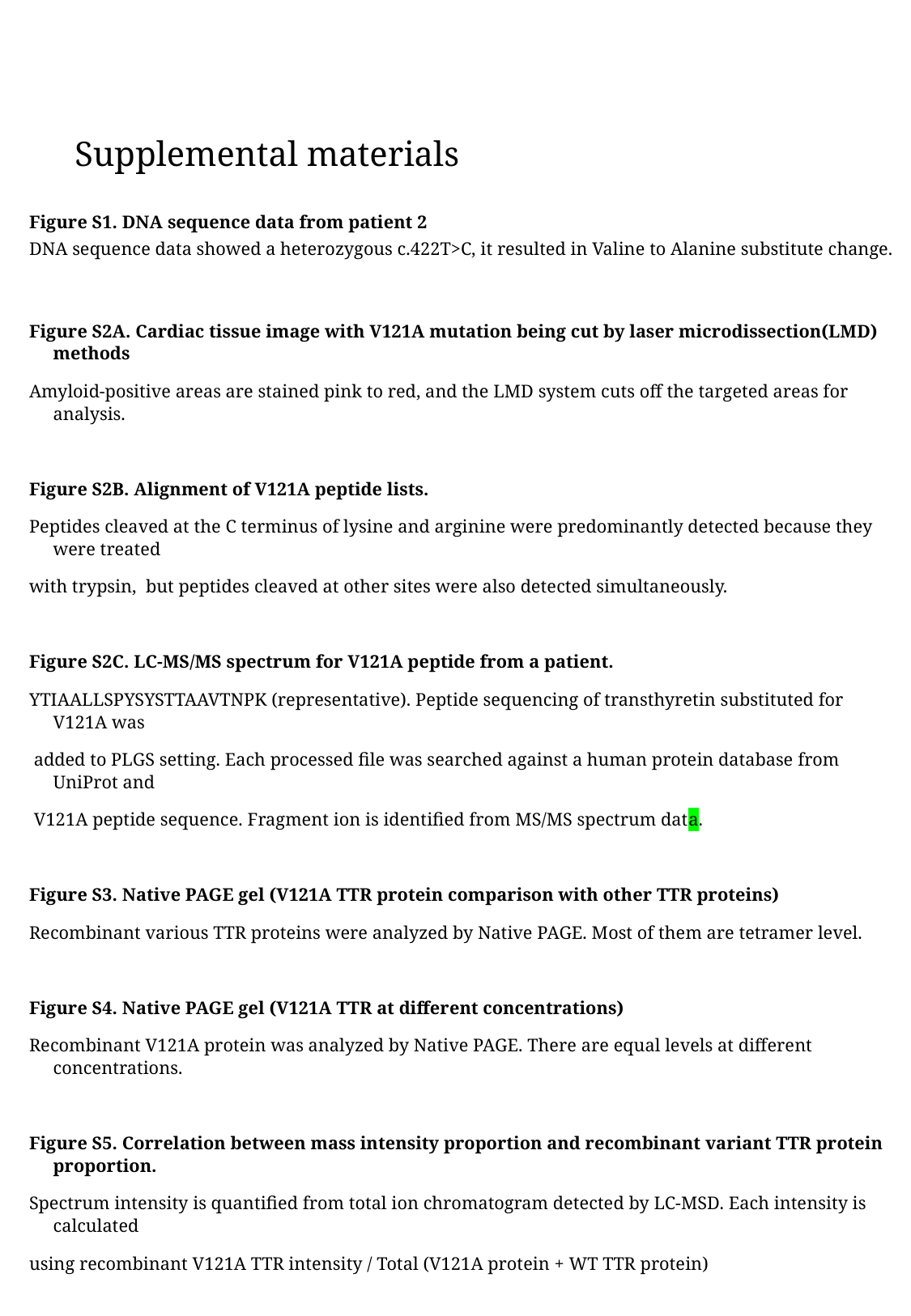

# Supplemental materials
Figure S1. DNA sequence data from patient 2
DNA sequence data showed a heterozygous c.422T>C, it resulted in Valine to Alanine substitute change.
Figure S2A. Cardiac tissue image with V121A mutation being cut by laser microdissection(LMD) methods
Amyloid-positive areas are stained pink to red, and the LMD system cuts off the targeted areas for analysis.
Figure S2B. Alignment of V121A peptide lists.
Peptides cleaved at the C terminus of lysine and arginine were predominantly detected because they were treated
with trypsin, but peptides cleaved at other sites were also detected simultaneously.
Figure S2C. LC-MS/MS spectrum for V121A peptide from a patient.
YTIAALLSPYSYSTTAAVTNPK (representative). Peptide sequencing of transthyretin substituted for V121A was
 added to PLGS setting. Each processed file was searched against a human protein database from UniProt and
 V121A peptide sequence. Fragment ion is identified from MS/MS spectrum data.
Figure S3. Native PAGE gel (V121A TTR protein comparison with other TTR proteins)
Recombinant various TTR proteins were analyzed by Native PAGE. Most of them are tetramer level.
Figure S4. Native PAGE gel (V121A TTR at different concentrations)
Recombinant V121A protein was analyzed by Native PAGE. There are equal levels at different concentrations.
Figure S5. Correlation between mass intensity proportion and recombinant variant TTR protein proportion.
Spectrum intensity is quantified from total ion chromatogram detected by LC-MSD. Each intensity is calculated
using recombinant V121A TTR intensity / Total (V121A protein + WT TTR protein)

## Slide 4
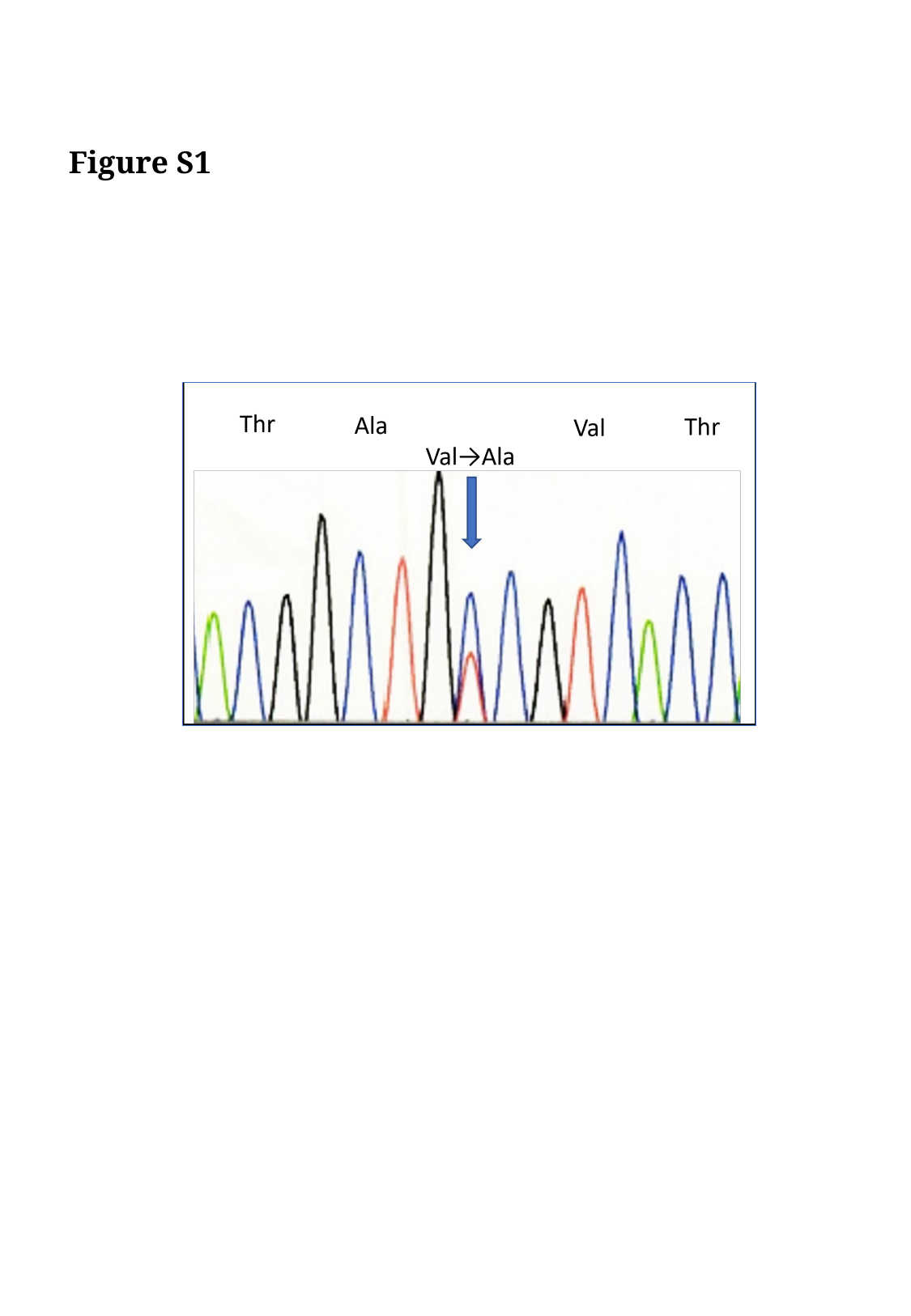

Figure S1

## Slide 5
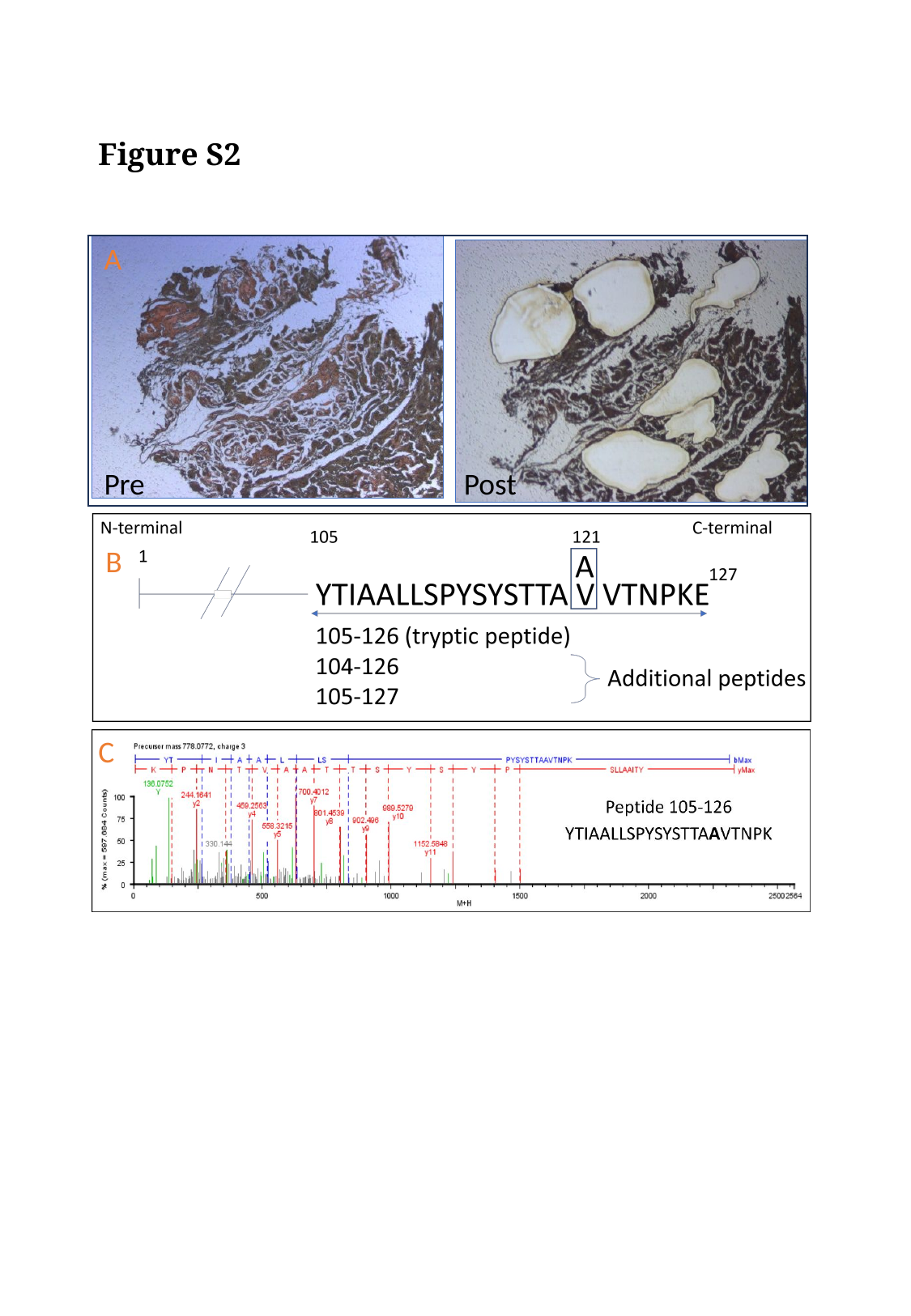

Figure S2
A
Pre
Post
B
C

## Slide 6
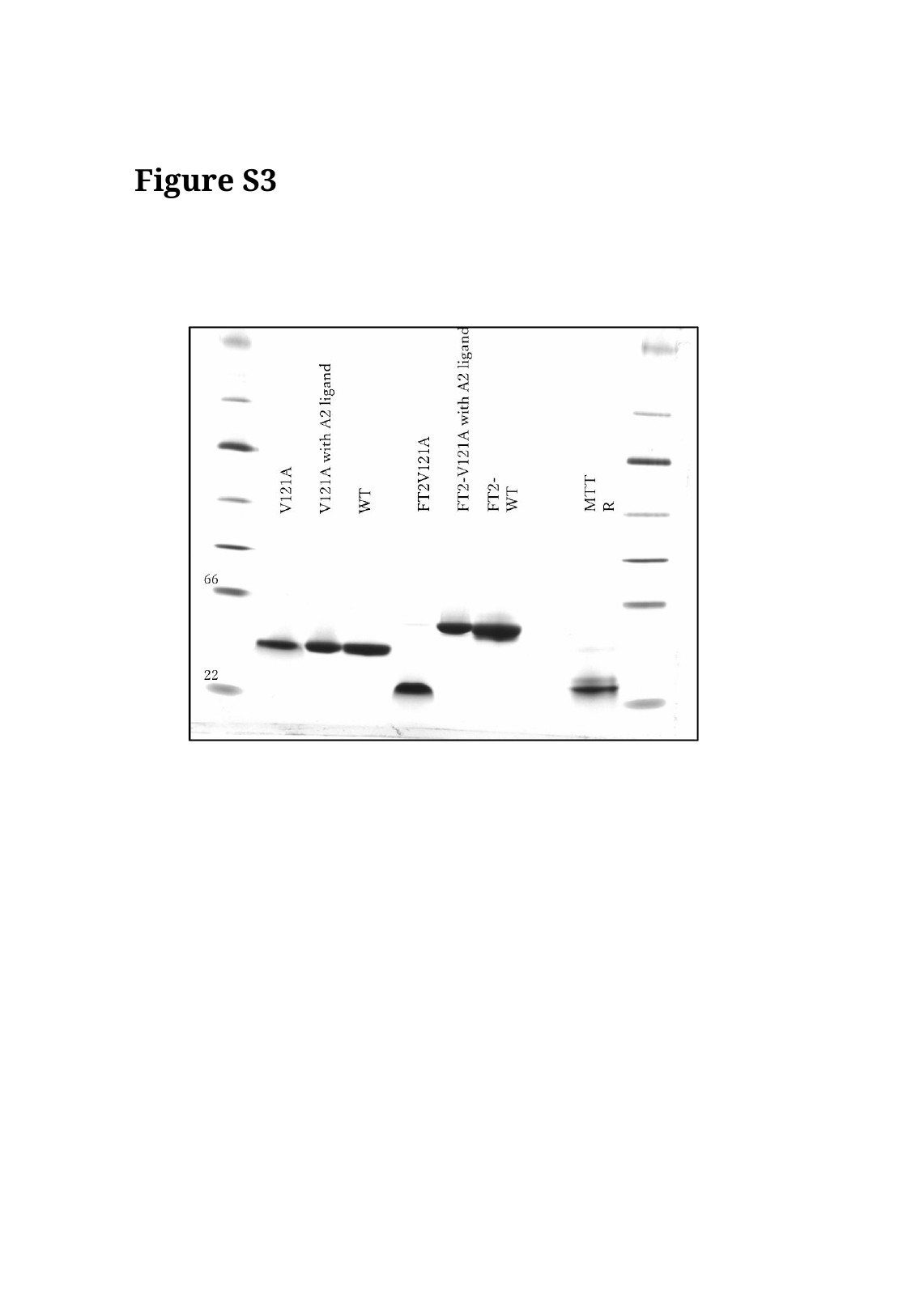

Figure S3

## Slide 7
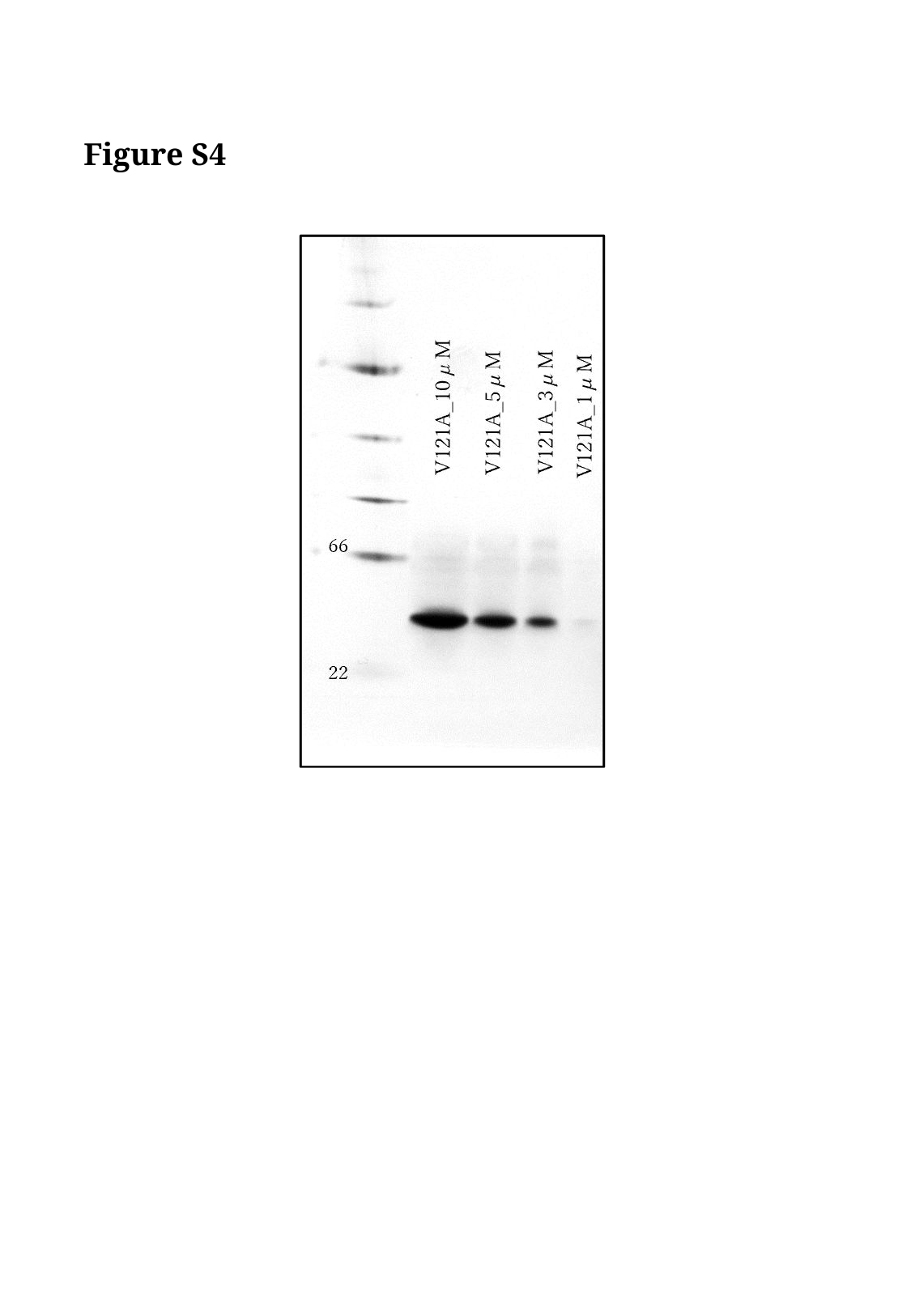

Figure S4

## Slide 8
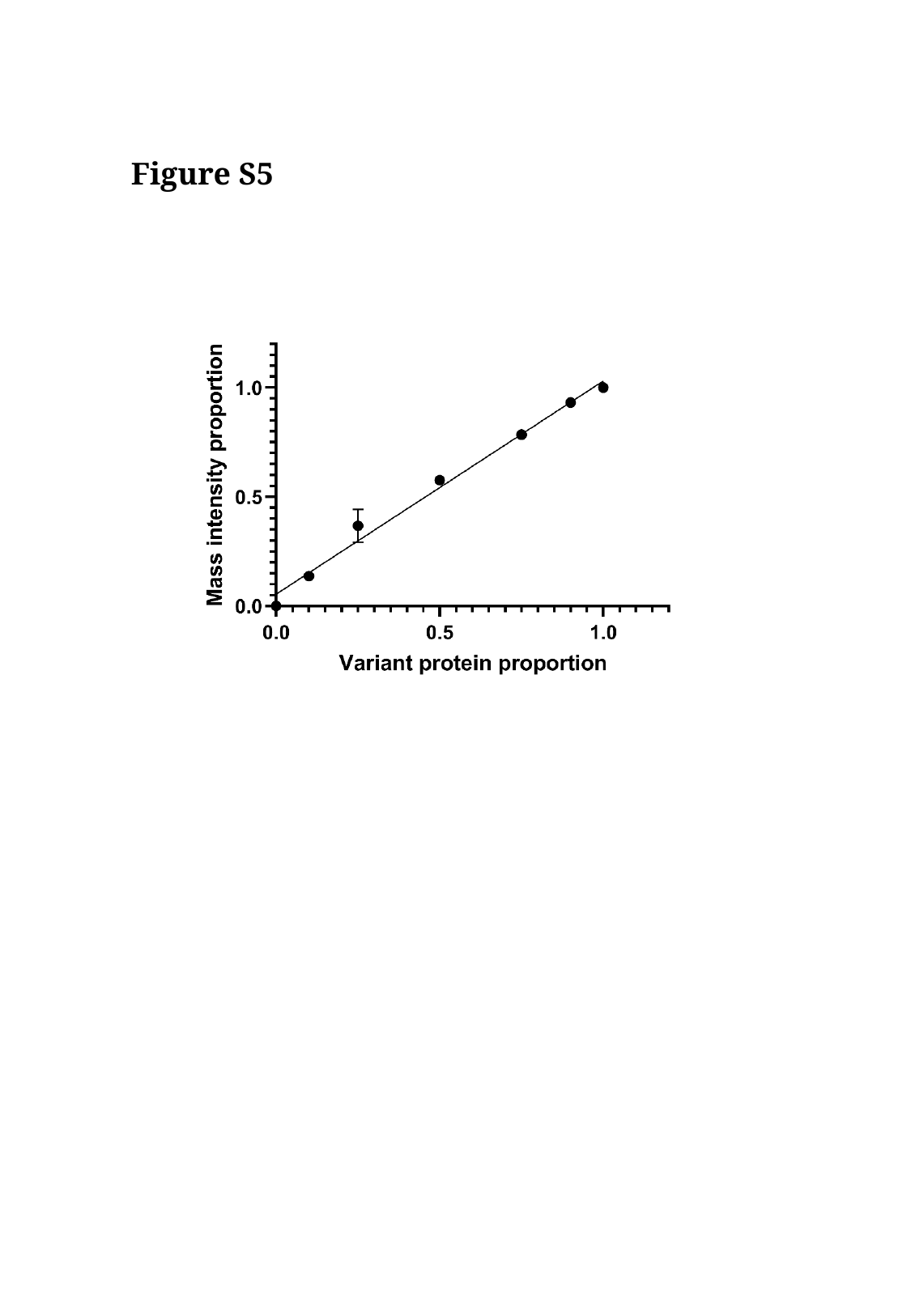

Figure S5
